# A simplified frailty scale predicts outcomes in transplant-ineligible patients with newly diagnosed multiple myeloma treated in the FIRST (MM-020) trial

**DOI:** 10.1038/s41375-019-0539-0

**Published:** 2019-08-19

**Authors:** Thierry Facon, Meletios A. Dimopoulos, Nathalie Meuleman, Andrew Belch, Mohamad Mohty, Wen-Ming Chen, Kihyun Kim, Elena Zamagni, Paula Rodriguez-Otero, William Renwick, Christian Rose, Adrian Tempescul, Eileen Boyle, Salomon Manier, Michel Attal, Philippe Moreau, Margaret Macro, Xavier Leleu, Marie Lorraine Chretien, Heinz Ludwig, Shien Guo, Michael Sturniolo, Antoine Tinel, Mara Silvia Monzini, Bruno Costa, Vanessa Houck, Cyrille Hulin, Jean Yves Mary

**Affiliations:** 1 Univ.Lille, CHU Lille, Service des Maladies du Sang, F-59000 Lille, France; 2 National and Kapodistrian University of Athens, Athens, Greece; 3 Jules Bordet Institute, Université Libre de Bruxelles, Brussels, Belgium; 4 Cross Cancer Institute, Edmonton, AB Canada; 5 Department of Haematology, Saint Antoine Hospital, Paris, France; 6 Department of Hematology, Beijing Chaoyang Hospital, Capital Medical University, Beijing, China; 7 Sungkyunkwan University Samsung Medical Center, Seoul, Korea; 8 Azienda Ospedaliero-Universitaria, Malpighi, Bologna, Italy; 9 University of Navarra, Pamplona, Spain; 10 Western Health, Melbourne, Australia; 11 Hôpital Saint Vincent de Paul Université Catholique de Lille, Lille, France; 12 Centre Hospitalier Universitaire, Brest, France; 13 Institut Universitaire du Cancer Toulouse Oncopole, Toulouse, France; 14 University Hospital Hôtel-Dieu, Nantes, France; 15 Centre Hospitalier Universitaire, Caen, France; 16 Hôpital La Mileterie, Poitiers, France; 17 CHU de Dijon, Dijon, France; 18 Wilhelminen Hospital, Vienna, Austria; 19 Evidera, Waltham, MA, USA; 20 Celgene Corporation, Summit, NJ USA; 21 Celgene International Sàrl, Boudry, Switzerland; 22 Centre Hospitalier Universitaire, Bordeaux, France; 23 Hôpital Saint-Louis, Paris, France

**Keywords:** Haematological diseases, Clinical trials

## Abstract

Patients with multiple myeloma are generally older and vary in fitness levels, which may influence the clinical benefit of treatment. Patients from the large, phase 3 FIRST trial in newly diagnosed multiple myeloma (NDMM) were retrospectively investigated to determine outcomes based on frailty using scores for age, Charlson Comorbidity Index (CCI), and Eastern Cooperative Oncology Group performance status (ECOG PS), instead of the EQ-5D quality-of-life questionnaire, as previously reported. ECOG PS (*n* = 1618) was investigated in frailty groups: frail (49%) and nonfrail (51%). Frail patients experienced worse progression-free and overall survival vs nonfrail patients. Prognostic assessment was improved when combining frailty and International Staging System stage (I/II vs III). Frail patients had a higher risk of developing grade 3/4 treatment-emergent adverse events. Treatment effects observed in the FIRST trial were confirmed per frailty group and per frailty and ISS group. The use of this ECOG PS–containing frailty scale as a predictive measure of clinical outcomes in patients with transplant-ineligible NDMM is supported by data from the FIRST trial. This score, based on age, CCI, and ECOG PS, can be easily replicated and may help design future myeloma studies in frail or nonfrail elderly patients.

## Introduction

Multiple myeloma is predominantly a disease of the elderly, with a median age of 69 years at diagnosis [1]. Treatment with newer agents has improved survival outcomes for patients with newly diagnosed multiple myeloma (NDMM) [2, 3], especially among those >65 years of age [3]. However, elderly patients are a heterogeneous population that varies greatly in fitness levels, with frail patients often underrepresented in clinical trials. Varying fitness levels may affect the time on treatment and how well a given regimen is tolerated, which could influence clinical benefit [4]. Currently, there is strong interest in developing a robust frailty scale that could improve treatment decisions for elderly patients with myeloma and help design specific studies in frail or nonfrail patients. However, developing a frailty scale remains challenging because it needs to perform better than current standards, not be subjective, and be convenient for physicians. Theoretically, considering that age and comorbidities may be part of any frailty scoring system, the choice between a patient-derived frailty assessment using geriatric questionnaires vs a physician-derived frailty assessment needs to be made, with the former typically considered more consistent [5].

Recently, the International Myeloma Working group (IMWG) introduced a scoring system to classify the frailty of elderly patients based on age, comorbidities (Charlson Comorbidity Index; CCI), and patient-evaluated self-care and household management assessments using the Katz Activity of Daily Living (ADL) [6] and Lawton Instrumental Activity of Daily Living (IADL) scales, respectively [4, 7]. The IMWG frailty scale classified patients as fit, intermediate, or frail, and these classifications were able to predict survival and risk of toxicity from treatment in patients with NDMM [4]. The IMWG study included some transplant-ineligible patients treated with lenalidomide and dexamethasone, which is now the standard treatment for this patient group [8]. The combination of this frailty scale with the International Staging System (ISS) [9] increased its prognostic value [4]. A separate study validated the use of the IMWG score when compared with other comorbidity scores commonly used in clinical practice [10]. Other studies have explored frailty scoring in patients with NDMM, including one that used the N-terminal fragment of the B-type natriuretic peptide to determine frailty, finding it was a useful predictor of survival [11].

The Frontline Investigation of Revlimid and Dexamethasone Versus Standard Thalidomide combination therapy (FIRST) trial (MM-020) is the largest phase 3 trial conducted in elderly patients with NDMM [12, 13]. The trial assessed continuous lenalidomide and dexamethasone (Rd continuous) vs lenalidomide and dexamethasone for 18 cycles (Rd18) vs fixed-duration melphalan + prednisone + thalidomide (MPT) [12]. The study demonstrated that Rd continuous significantly prolonged progression-free survival (PFS) and overall survival (OS) compared with MPT, and significantly prolonged PFS compared with Rd18 [13]. The study also incorporated a comprehensive quality-of-life analysis using the European Organisation for Research and Treatment of Cancer QLQ-C30, myeloma QLQ-MY20, and EQ-5D questionnaires [14]. However, the FIRST trial did not use the ADL and IADL scales used in the IMWG score. Therefore, the initial frailty analysis included the EQ-5D questionnaire (three levels, five dimensions), a standardized measure of health status designed by the EuroQol Group to be completed by the patient, as a proxy for the ADL and IADL scales [15, 16]. The initial analysis categorized the 1517 patients with baseline EQ-5D assessments in the intent-to-treat (ITT) population as fit (*n* = 255; 17%), intermediate (*n* = 448; 30%), or frail (*n* = 814; 54%) [15]. Fit patients had improved PFS and OS compared with intermediate fitness and frail patients. In addition, the analysis showed that Rd continuous had PFS and OS benefits compared with MPT regardless of frailty level, with fit patients demonstrating the greatest benefits.

Subsequently, Eastern Cooperative Oncology Group (ECOG) performance status, a physician assessment of a patient’s level of functioning in terms of self-care, daily activity, and physical ability, was investigated. Demonstrating the prognostic value of a frailty score using ECOG performance status could be beneficial, as it is commonly used in clinical trials and can be easily used in clinical practice [5]. This analysis classified patients into frail and nonfrail subgroups and examined outcomes based on this classification.

## Patients and methods

This is a subanalysis of the multicenter, open-label, phase 3 FIRST trial (MM-020/IFM07-01; NCT00689936) to determine outcomes in patients based on frailty. The study design and patient population of the FIRST trial have been previously reported [12]. In brief, patients must have had previously untreated, symptomatic, and measurable multiple myeloma, as defined by IMWG criteria [17], and been ineligible for stem cell transplant (either ≥65 years of age or <65 years of age and ineligible for transplant), with an ECOG performance status ≤ 2. The primary comparators in the FIRST trial were Rd continuous vs MPT.

Patients were divided into frailty categories using baseline patient characteristics including age, CCI (based on the medical history of all reported patients), and ECOG performance status, instead of the EQ-5D quality-of-life questionnaire, as previously reported [15]. A frailty categorization based on ECOG performance status was investigated with three categories (frail, intermediate fitness, and fit), which facilitated a comparison of the ECOG and EQ-5D scoring systems. Subsequently, the ECOG-based frailty assessment was simplified by using only two categories (nonfrail, 0–1; frail, ≥2; Table 1). Patients within each frailty group were further divided by ISS stage (I/II vs III) to define frailty and ISS groups. Patients with missing data on ≥1 variable were excluded (*n* = 5).

**Table 1:** ECOG proxy of IMWG algorithm of frailty

Category	Score
Age	
≤75 years	0
76–80 years	1
>80 years	2
Charlson Comorbidity Index	
≤1	0
>1	1
ECOG performance status	
0	0
1	1
≥2	2
Sum of scores	
Nonfrail	0–1
Frail	≥2

*ECOG* Eastern Cooperative Oncology Group, *IMWG* International Myeloma Working Group

Agreement between frailty scores derived from EQ-5D and ECOG was assessed through proportions of disagreements and kappa statistic. The ability of the two frailty scoring systems to predict OS could not be compared directly. Indeed, when the OS curve for the 778 frail patients derived from the EQ-5D questionnaire is compared with that of the 739 frail patients derived from ECOG performance status, 587 patients belong to both curves since they were considered frail according to the two frailty scoring systems, whereas 191 patients among the 778 EQ-5D frail patients belong to only 1 curve and 152 patients among the 739 ECOG frail patients belong to only the other curve. Thus, a comparison of these two curves (i.e., OS curve based on 778 frail patients from the EQ-5D scoring system and 739 frail patients from the ECOG scoring system) is not straightforward. This is also true when dealing with nonfrail patients. Our solution was to study the four OS curves defined by the two frailty assessments in patients defined as: frail by both scores, frail by EQ-5D and nonfrail by ECOG, nonfrail by EQ-5D and frail by ECOG, and nonfrail by both scores. The curves derived from patients who were frail by both scores or nonfrail by both scores did not provide any information about the differences in OS by frailty scoring systems. Contrarily, the two intermediate curves, frail by EQ-5D and nonfrail by ECOG, and nonfrail by EQ-5D and frail by ECOG, do provide information, and their comparison through the log-rank test and Cox model allows us to test whether a difference in OS does exist between the two scoring systems.

The remainder of the methodology is based on the frailty assessment using ECOG performance status. Comparison of response rates according to frailty group was performed through the chi-square test. Proportions of patients having not experienced an event of interest over time after randomization were estimated through the Kaplan–Meier method. The events of interest examined in the analyses included death, progression or death without progression, treatment discontinuation, and first grade 3/4 treatment-emergent adverse event (TEAE) occurrence, each of which was analyzed separately. In the analyses of time to treatment discontinuation and time to first grade 3/4 TEAE occurrence, patients were censored at the time of progression, death, or end of follow-up (i.e., completion of expected treatment cycles for the former and end of treatment plus 28 days for the latter), whichever occurred first. Comparison of time-to-event curves according to frailty group and according to frailty and ISS group was performed through a Cox proportional hazard model, with results expressed as the hazard ratio (HR). An HR >1 indicates a worse outcome in the studied group compared with the control group, i.e., an increased risk of progression or death without progression for PFS and an increased risk of death for OS. To examine whether the relative effects of frailty group on these outcomes of interest vary by treatment group, the interaction was tested through the likelihood ratio test, either in a logistic model (response) or Cox proportional hazard model (time to event). Comparison of the OS and PFS prognostic assessments per frailty group and per frailty and ISS group was performed through the likelihood ratio test. All the analyses were performed using data with a cutoff date of January 21, 2016, which had a median follow-up duration of 5.6 years.

## Results

### Patient characteristics

All data, including baseline ECOG performance status, were available for 1618 of the 1623 patients from the ITT population of the FIRST trial. Of this population, 49% of patients (*n* = 790) were in the frail cohort. The median age of frail patients was higher and a greater proportion had ISS stage III disease, elevated lactate dehydrogenase levels (≥200 U/L), and worse renal function (creatinine clearance <60 mL/min) at baseline compared with nonfrail patients (Table 2).

**Table 2:** Baseline characteristics by frailty group

Characteristic	Nonfrail (*n* = 828)	Frail (*n* = 790)
Age, *n* (%)		
Median (range), years	70 (40–80)	77 (44–92)
<65 years	66 (8)	26 (3)
65–75 years	754 (91)	299 (38)
76–80 years	74 (9)	290 (37)
>80 years	0	201 (25)
Sex, *n* (%)		
Male	436 (53)	415 (53)
Female	392 (47)	375 (47)
ECOG performance status, *n* (%)^a^		
0	401 (48)	73 (9)
1	427 (52)	368 (47)
2	0	343 (43)
3	0	6 (<1)
Data not available	0	0
International Staging System stage, *n* (%)^b^		
I or II	575 (69)	419 (53)
III	253 (31)	371 (47)
Lactate dehydrogenase, *n* (%)		
<200 U/L	707 (85)	612 (78)
≥200 U/L	120 (15)	177 (22)
Missing data	1	1
Creatinine clearance, *n* (%)		
<60 mL/min	299 (36)	478 (61)
<30 mL/min	38 (5)	108 (14)
≥60 mL/min	529 (64)	312 (39)

*ECOG* Eastern Cooperative Oncology Group

^a^ECOG scores range from 0 to 5, with higher numbers indicating greater disability

^b^Higher stages indicate more severe disease

### Comparison of EQ-5D and ECOG and rationale for using ECOG

**Fig. 1:**
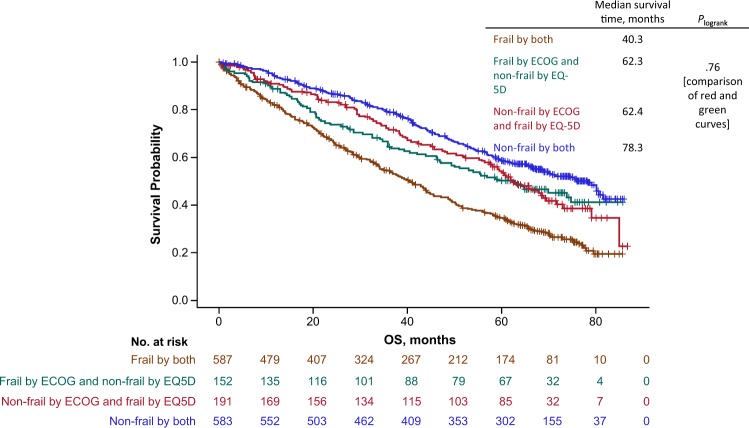
OS by frailty score using either the EQ-5D questionnaire or ECOG performance status. OS Overall Survival, ECOG Eastern Cooperative Oncology Group

The frailty scores using the EQ-5D questionnaire and ECOG performance status classify patients differently, with the former using both self-care and usual activities scores, and the latter using only the overall ECOG score. In a comparative analysis, 65% of patients were classified in the same frailty group, 32% had a 1-level difference, and 3% had a 2-level difference, leading to a kappa value of 0.43 (Table 3). The four OS curves were defined by the two frailty assessments with EQ-5D and ECOG (Fig. 1). Not surprisingly, shorter survival is observed in patients defined as frail by both scoring systems, while longer survival is observed in patients defined as nonfrail by both scoring systems. However, even if the curve observed in patients defined as frail by ECOG and nonfrail by EQ-5D seems to be less favorable than the curve of patients defined as nonfrail by ECOG and frail by EQ-5D, no significant difference between these two OS curves could be evidenced (*P* = 0.72). Based on this and the ease of calculation, we used only the ECOG-containing frailty score in the forthcoming analyses.

**Table 3:** Comparison of frailty group classifications when using EQ-5D and ECOG performance status in addition to Charlson Comorbidity Index and age

	EQ-5D			
ECOG	Fit	Intermediate	Frail	Total
Fit	154	72	26	252
Intermediate	113	244	165	522
Frail	13	139	587	739
Total	280	455	778	1513

*ECOG* Eastern Cooperative Oncology Group

### Frailty analysis

#### Outcomes by frailty group

**Fig. 2:**
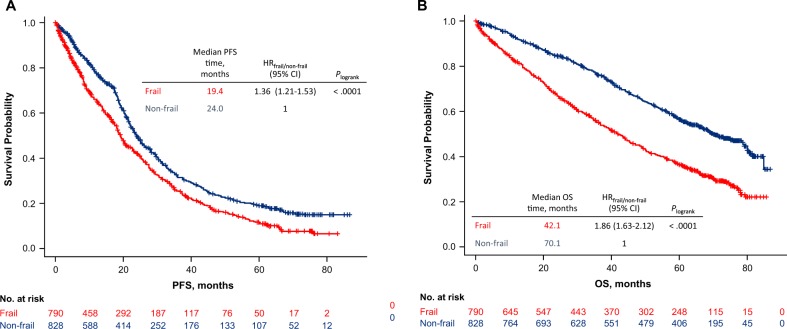
PFS (**a**) and OS (**b**) by frailty group. PFS Progression-free Survival, OS Overall Survival

Overall response rate was significantly lower in the frail group compared with the nonfrail group (72 vs 79%; *P* = 0.0002; Supplementary Table 1). This effect did not vary across treatment groups (interaction test, *P* = 0.73). Frail patients also experienced a worse PFS compared with nonfrail patients (median PFS, 19.4 vs 24.0 months; HR = 1.36; 95% CI, 1.21–1.53; *P* < 0.0001; Fig. 2a). The variation of PFS according to frailty group did not vary across treatment groups (interaction test, *P* = 0.18). The 12- and 18-month PFS rates were 65% and 54%, respectively, in frail patients, and 78% and 68%, respectively, in nonfrail patients. Frail patients also experienced a worse OS compared with nonfrail patients (median OS, 42.1 vs 70.1 months; HR = 1.86; 95% CI, 1.63–2.12; *P* < 0.0001; Fig. 2b). The variation of OS according to frailty group did not vary across treatment groups (interaction test, *P* = 0.20). The 12-month OS rate was 82% in frail patients and 92% in nonfrail patients, while the 18-month OS rate was 75% and 89% in frail and nonfrail patients, respectively.

#### Outcomes by frailty and ISS group

**Fig. 3:**
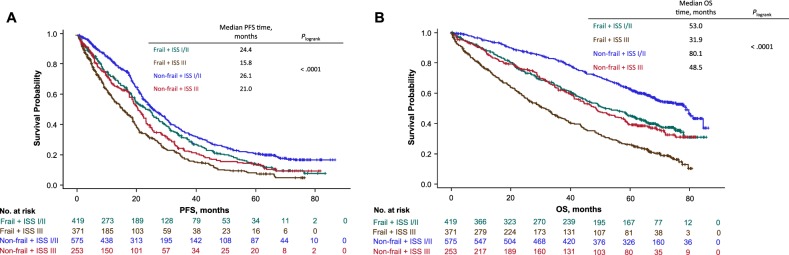
PFS (**a**) and OS (**b**) by frailty and ISS group. PFS Progression-Free Survival, OS Overall Survival, ISS International Staging System

Further subdividing both frailty groups by ISS stage improved the prognostic assessment across all severity groups for PFS (*P* < 0.0001) and OS (*P* < 0.0001) (Fig. 3a, b). The effect of frailty and ISS group on PFS and OS did not vary across treatment groups (interaction test, *P* = 0.54 and *P* = 0.13 for PFS and OS, respectively).

### Treatment discontinuation and safety by frailty group

**Fig. 4:**
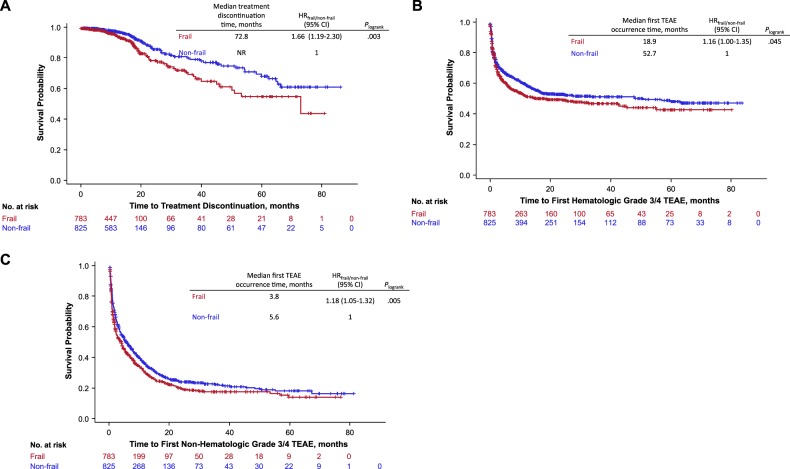
Time to treatment discontinuation (**a**) and first hematologic (**b**) and nonhematologic (**c**) TEAEs by frailty group. TEAE Treatment-Emergent Adverse Event

Time to premature discontinuation not due to disease progression or death was shorter for frail patients compared with nonfrail patients (HR = 1.66; 95% CI, 1.19–2.30; *P* = 0.003; Fig. 4a). The variation of treatment discontinuation according to frailty group did not vary across treatment groups (interaction test, *P* = 0.37). At 12 months, 95% of frail and 98% of nonfrail patients remained on treatment; 87% of frail and 93% of nonfrail patients remained on treatment at 18 months.

Compared with nonfrail patients, frail patients had a higher risk of developing first grade 3/4 hematologic TEAEs (HR = 1.16; 95% CI, 1.00–1.35; *P* = 0.045; Fig. 4b; Supplementary Table 2). This effect did not vary across treatment groups (interaction test, *P* *=* 0.62). The percentage of frail and nonfrail patients who had not experienced grade 3/4 hematologic TEAEs was 52% and 60%, respectively, at 12 months, and 50% and 54%, respectively, at 18 months.

Frail patients were also more likely than nonfrail patients to develop first grade 3/4 nonhematologic TEAEs (HR = 1.18; 95% CI, 1.05–1.32; *P* = 0.005; Fig. 4c). This effect did not vary across treatment groups (interaction test, *P* = 0.97). A total of 30% of frail and 35% of nonfrail patients had not experienced grade 3/4 nonhematologic TEAEs at 12 months; 24% and 27%, respectively, at 18 months. The risk analyses for developing TEAEs by frailty group and treatment arm are presented in the Supplementary Appendix.

### Treatment effect by frailty group and by frailty and ISS group

**Fig. 5:**
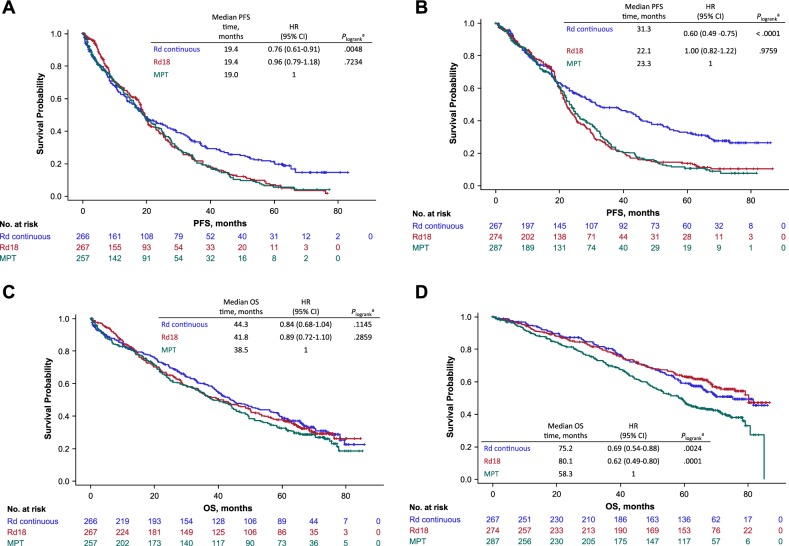
PFS by treatment group in frail patients (**a**) and nonfrail patients (**b**), and OS by treatment group in frail patients (**c**) and nonfrail patients (**d**) ^a^P values compare with MPT. PFS Progression-Free Survival, OS Overall Survival, MPT Melphalan + Prednisone + Thalidomide

**Fig. 6:**
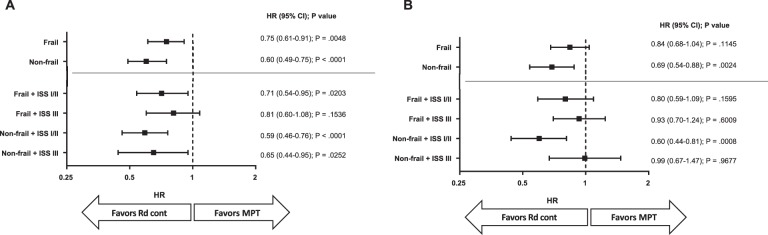
Comparison of Rd continuous vs MPT for PFS (**a**) using frailty subgroups with and without ISS stage, and comparison of Rd continuous vs MPT for OS (**b**) using frailty subgroups with and without ISS stage. MPT Melphalan + Prednisone + Thalidomide, PFS Progression-Free Survival, ISS International Staging System, OS Overall Survival

Within each treatment arm, patients were evenly divided as either frail or nonfrail. The proportion of frail patients was 50% in the Rd continuous group (*n* = 533), 49% in the Rd18 group (*n* = 541), and 47% in the MPT group (*n* = 544). Similar to the results in the ITT population, Rd continuous prolonged PFS and OS compared with MPT for both frail and nonfrail patients, with the greatest numerical benefit in nonfrail patients **(**Figs. 5 and 6). Indeed, the median PFS time was 19.4 vs 19.0 months (HR = 0.75; 95% CI, 0.61–0.91; *P* = 0.005) and the median OS time was 44.3 vs 38.5 months (HR = 0.84; 95% CI, 0.68–1.04; *P* = 0.11) in frail patients, while the median PFS time was 31.3 vs 23.3 months (HR = 0.60; 95% CI, 0.49–0.75; *P* < 0.0001) and the median OS time was 75.2 vs 58.3 months (HR = 0.69; 95% CI, 0.54–0.88; *P* = 0.002) in nonfrail patients. Analyses of PFS and OS according to frailty group by treatment arm are presented in the Supplementary Appendix (Supplementary Figs. 1 and 2).

The distribution of the number of patients per frailty and ISS group was similar across treatment arms (Supplementary Fig. 3) and the distribution of patient characteristics in these groups is described in Supplementary Table 3. Rd continuous prolonged both PFS and OS compared with MPT in most frailty and ISS groups, with the greatest numerical benefit in nonfrail + ISS I/II patients (PFS: HR = 0.59; 95% CI, 0.46–0.76; *P* < 0.0001; Fig. 6 and Supplementary Fig. 4; OS: HR = 0.60; 95% CI, 0.44–0.81; *P* = 0.0008; Fig. 6 and Supplementary Fig. 5). Outcomes by treatment arm are presented per frailty and ISS group in the Supplementary Appendix (Supplementary Figs. 6 and 7). Median dose intensity for lenalidomide was lower in frail patients compared with nonfrail patients (Supplementary Table 4).

## Discussion

The IMWG frailty scale published in 2015 by Palumbo et al. was influential work, drawing the attention of the myeloma community to the need for a robust frailty assessment; however, there is evidence that this score is not widely used in routine clinical practice. Others have attempted to establish alternative frailty scores [18] that may be easier to calculate with assessments commonly performed in clinical practice [10, 11, 18, 19]. One feature of the IMWG frailty scale is that it assumes that what is reported by the patient is more useful than the physician’s assessment. Another characteristic of the IMWG frailty scale is that it classifies patients in three categories: fit, intermediate fitness, and frail. However, having only two categories (nonfrail and frail), as opposed to three, would be more relevant from a clinical point of view and easier to manage for the design and analysis of clinical studies dedicated to frail patients. Interestingly, the frailty score used in this manuscript separates two almost equal populations of nonfrail and frail patients (48.8% frail).

Similar to the IMWG frailty scale [4], the frailty scale described here analyzed transplant-ineligible patients with NDMM and was able to predict PFS and OS outcomes. Frail patients experienced a worse PFS (*P* < 0.0001) and OS (*P* < 0.0001) compared with nonfrail patients. Subdividing fitness categories by ISS stage at diagnosis increased the prognostic value of the frailty scale, which is consistent with what was observed with the IMWG frailty scale. While most frailty and ISS groups trended toward PFS and OS benefit with Rd continuous vs MPT, nonfrail + ISS I/II patients had the greatest numerical benefit. In addition, while frail + ISS III patients showed a numerically lower median PFS compared with nonfrail + ISS III patients, the treatment effect in these patient groups showed Rd continuous was favored vs MPT. Similar to the safety results reported with the IMWG frailty scale, frail patients had a higher risk of grade 3/4 nonhematologic TEAEs compared with nonfrail patients; however, differing from the IMWG frailty scale results, frail patients had a higher risk of grade 3/4 hematologic TEAEs compared with nonfrail patients. Differences in the safety results reported with the IMWG frailty scale vs the frailty scale described here may be due to differences in the therapies used and data sets (i.e., the IMWG analysis pooled three trials with a total of 869 patients, whereas our analysis used a single large trial with a total of 1618 patients). For example, in the IMWG analysis, 24% of patients received proteasome inhibitor–containing regimens, whereas the majority of the FIRST patients received the immunomodulatory drug lenalidomide and only one-third received an alkylating agent [4].

As ECOG performance status represents an assessment of a patient’s level of functioning in terms of self-care, daily activity, and physical ability, it can be compared with the ADL and IADL scales. The ECOG scale directly provides one score according to general descriptions of patient performance status, whereas the ADL and IADL scales generate an aggregated score resulting from 6 to 8 items related to self-care or household management, which are scored individually. In addition, ECOG performance status is assessed by physicians, while the ADL and IADL scales are questionnaires filled out by patients. Studies have shown the association between patient and physician assessments for ECOG performance status [5], although a previous study has shown that ECOG may be improved with functional assessment of elderly cancer patients [20], and there is debate as to the benefit of patient- vs physician-derived scales [21]. Despite these differences, we do not expect the classification of patients into frailty groups to vary widely between the two analyses given the simplified cutoffs used in the final stratification of variables. Indeed, we initially conducted an analysis using the patient-assessed EQ-5D questionnaire, categorizing patients into three severity groups, as in the IMWG score (fit, intermediate, and frail) [15]. That analysis [15] was compared with the ECOG-based score. The two scores were not equivalent, showing a 35% discrepancy in ≥1 frailty level and a 3% discrepancy of two levels (i.e., fit vs frail). This may be a result of the narrow groupings of the 3-category scale (fit = 0, intermediate = 1, and frail = ≥2), in which a slight difference in the score could impact the final frailty assessment. For example, the presence of two EQ-5D components factored into the overall score (vs a single ECOG value) may partially account for some of this discrepancy. Overall, however, the OS prediction did not demonstrate a significant difference between the ECOG and EQ-5D scores. The combination of ECOG and EQ-5D could be attractive because it combines a patient’s assessment and a physician’s assessment, particularly with the approximately one-third of patients defined as frail by both who have a very poor prognosis. However, we believe that this age, comorbidity, and ECOG-containing scoring algorithm is much easier to use due to the balance of capturing relevant data without the need for complicated testing.

One limitation of this study is the absence of a validation data set or comparison between our frailty algorithm and other common algorithms derived by other groups using the same data set. This analysis was limited to a single clinical trial in which patients were predominately ECOG performance status ≤2 and two of the three arms contained the same treatments. In addition, we did not evaluate other patient populations, clinical trials, frailty measurements after baseline (as treatment could have improved frailty status), or patients outside of the clinical trial setting. Furthermore, our analysis did not incorporate the revised ISS criteria, as not all patients had cytogenetic analyses conducted. Finally, we have not explored patient-reported outcomes and quality-of-life assessments, which have shown some predictive value in other settings [22, 23].

This analysis of the FIRST trial population supports the use of an ECOG-containing frailty scale for predicting clinical outcomes in transplant-ineligible patients with NDMM. It is interesting to hypothesize that the use of a frailty scale based on age, comorbidities, and physical functioning could allow for better discrimination between elderly patients compared with evaluations based on age only. The prognostic ability of the frailty scale described here was demonstrated by further subdividing patients by ISS stage, and was a sensitive and easy-to-use predictor of survival. The results from these analyses reinforce the findings showing a benefit of Rd continuous over MPT regardless of fitness, with the greatest numerical benefit observed in nonfrail + ISS I/II patients. Future exploration of the frailty scale may be used to compare clinical trial populations of elderly patients, to design studies dedicated to elderly frail or nonfrail patients, and to implement risk-adapted treatment strategies for patients with multiple myeloma.

## Supplementary information

Supplemental Material
