# Supplementary material for: A simplified frailty scale predicts outcomes in transplant-ineligible patients with newly diagnosed multiple myeloma treated in the FIRST (MM-020) trial
Source: Leukemia. 2019 Aug 19;34(1):224–33. doi: 10.1038/s41375-019-0539-0 (PMC7214253; doi:10.1038/s41375-019-0539-0)
Supplement: Supplementary file 1 — Supplemental Material [file 41375_2019_539_MOESM1_ESM.docx]

**A simplified frailty scale predicts outcomes in transplant-ineligible patients with newly diagnosed multiple myeloma treated in the FIRST (MM-020) trial**

**Supplemental Appendix**

**Supplemental Results**

*Outcomes by treatment arm according to frailty group*

Compared with MPT, Rd continuous significantly reduced the risk of progression or death for both frail (HR = 0.75; 95% CI, 0.61-0.91; *P* = .005) and non-frail (HR = 0.60; 95% CI, 0.49-0.75; *P* < .0001) patients (**Figure 5A and B and Figure 6**).^[[1]](#endnote-2)^ Frail and non-frail patients experienced the greatest PFS benefit with Rd continuous compared with MPT. A 25% to 40% improvement in PFS was seen with Rd continuous vs MPT across both frailty groups, with the greatest benefit seen in non-frail patients.^[[2]](#endnote-3)^ For patients receiving Rd continuous, Rd18, or MPT, frail patients had significantly shorter median PFS time than non-frail patients (**Supplemental Figure 1A-C**).^[[3]](#endnote-4)^ Rd continuous reduced the risk of death vs MPT by 16% and 31% for frail and non-frail patients, respectively (**Figure 5C and D)**.^[[4]](#endnote-5)^ Rd18 also reduced the risk of death vs MPT by 38% for non-frail patients.^[[5]](#endnote-6)^ Within all treatment arms, frail patients experienced a worse OS benefit than non-frail patients (**Supplemental Figure 2A-C).^[[6]](#endnote-7)^**

*Safety by treatment arm according to frailty group*

Both frail and non-frail patients who received Rd continuous vs MPT had a lower risk of developing grade 3/4 hematologic TEAEs (HR = 0.54; 95% CI, 0.43-0.70; *P* < .0001; HR = 0.57; 95% CI, 0.44-0.74; *P* < .0001, respectively).^[[7]](#endnote-8)^ Patients treated with Rd continuous vs MPT had a similar risk of grade 3/4 non-hematologic TEAEs in both frail (HR = 1.03; 95% CI, 0.84-1.26; *P* = .796 and non-frail (HR = 1.03; 95% CI, 0.85-1.25; *P* = .772) patients.^[[8]](#endnote-9)^

**Supplemental Figure 1.** PFS by frailty group according to treatment arm: Rd continuous **(A)**, Rd18 **(B)**, and MPT **(C)**.^[[9]](#endnote-10)^

**
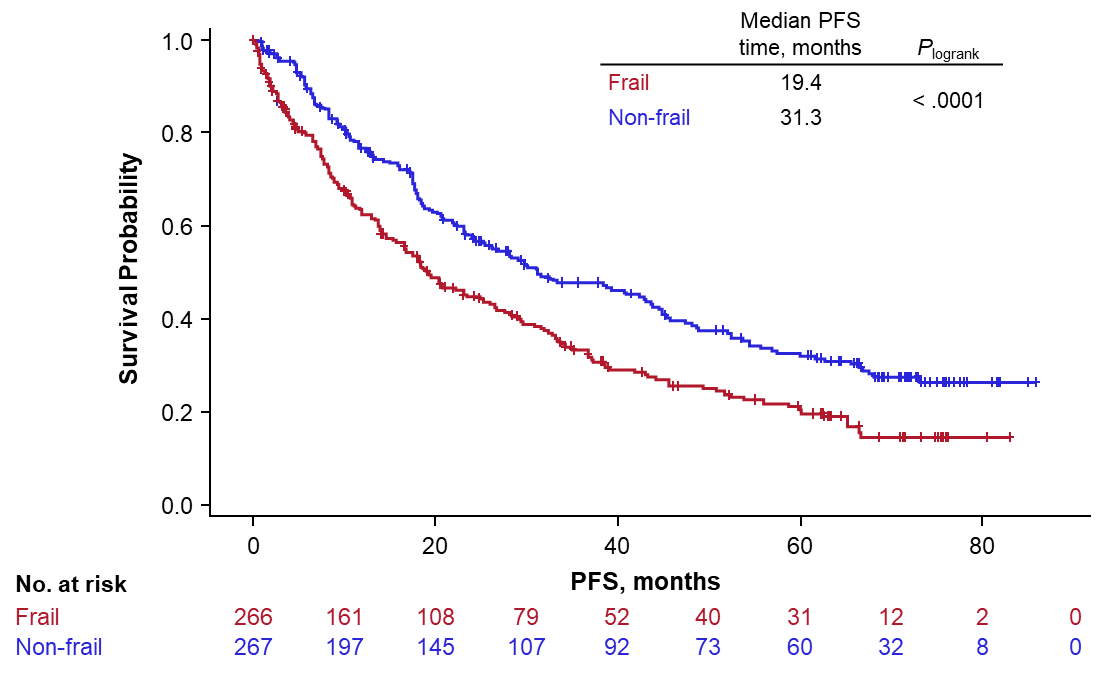
**
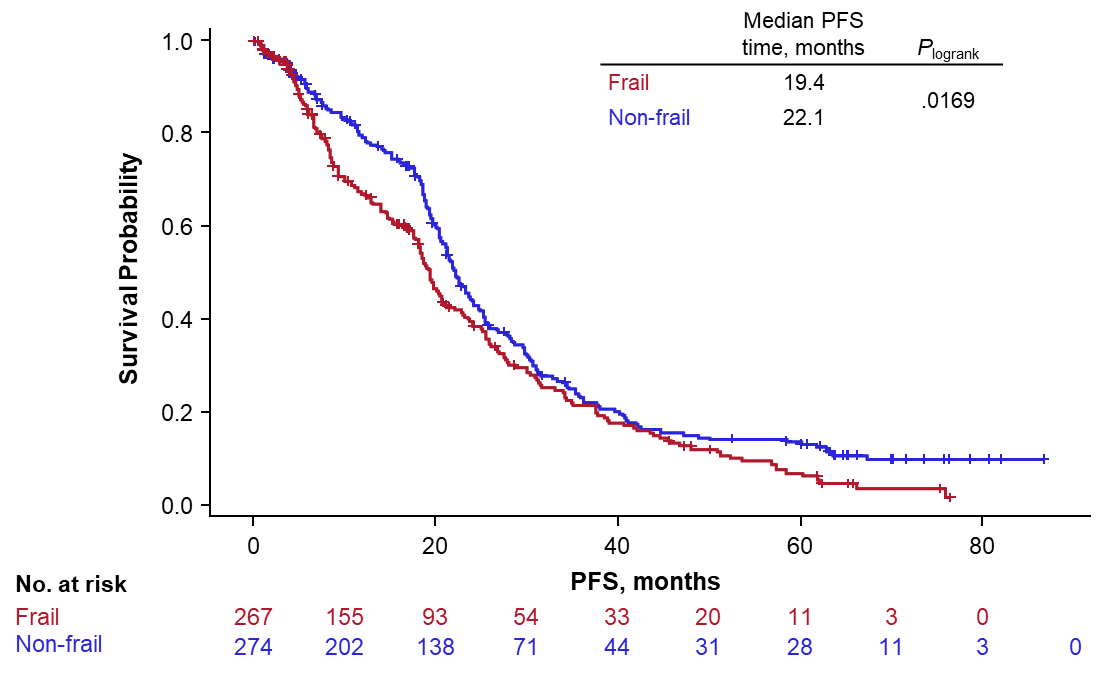
**A. B.**

**
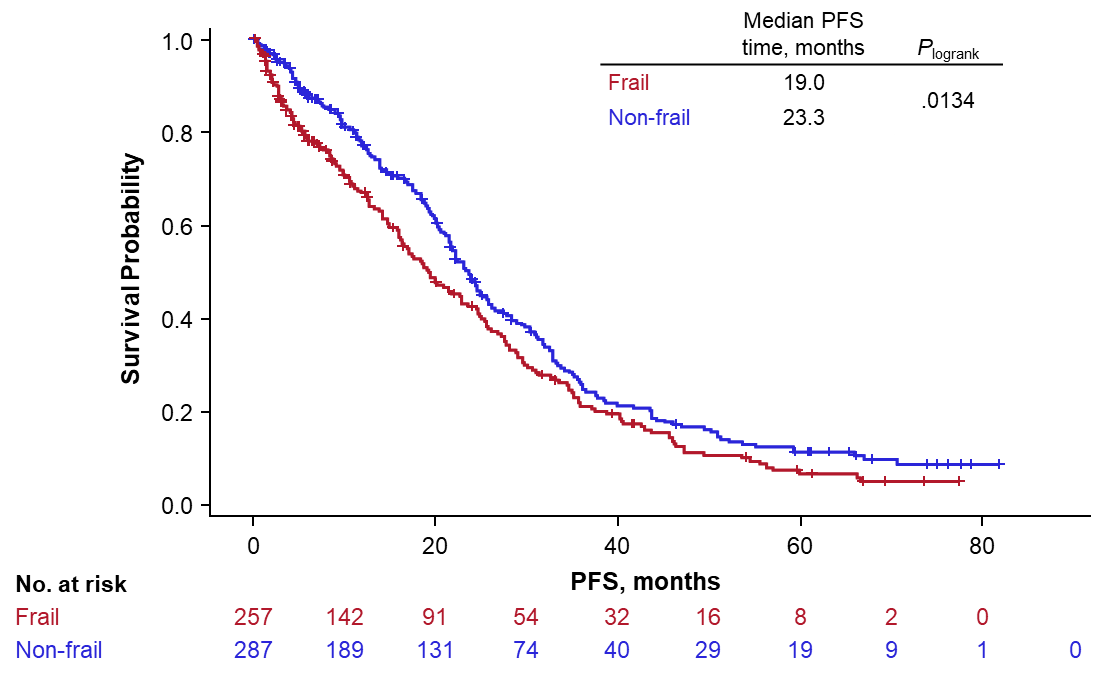
C.**

MPT, melphalan + prednisone + thalidomide; PFS, progression-free survival; Rd continuous, lenalidomide and low-dose dexamethasone until disease progression; Rd18, Rd for 18 cycles.

**Supplemental Figure 2.** OS by frailty group according to treatment arm: Rd continuous **(A)**, Rd18 **(B)**, and MPT **(C)**.^[[10]](#endnote-11)^

**
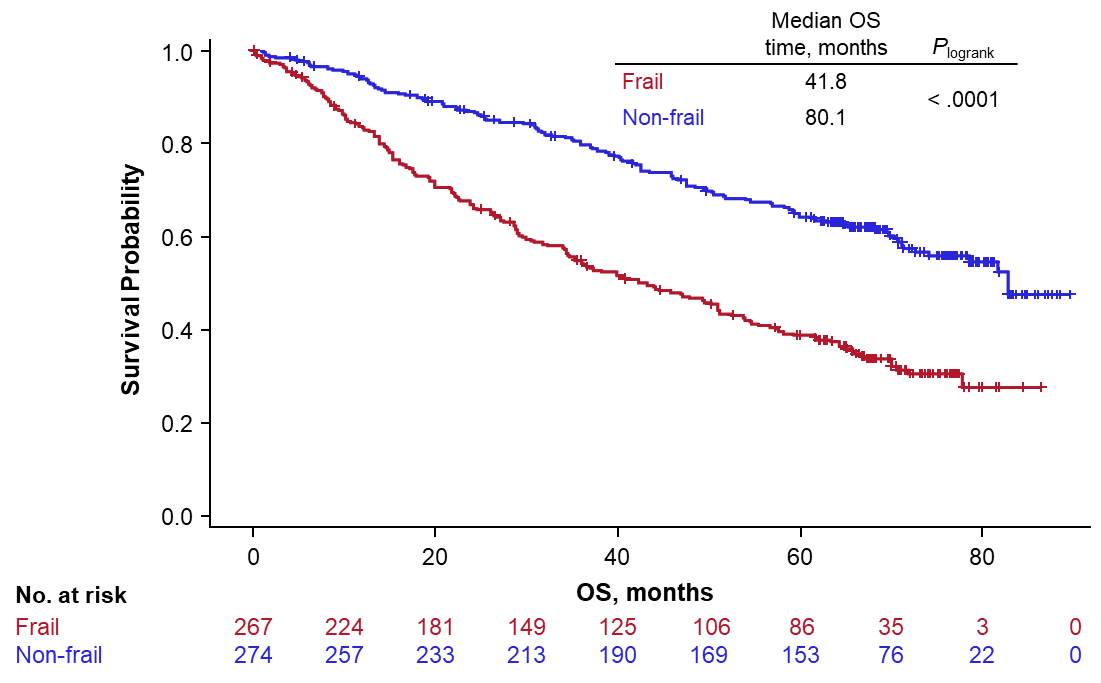

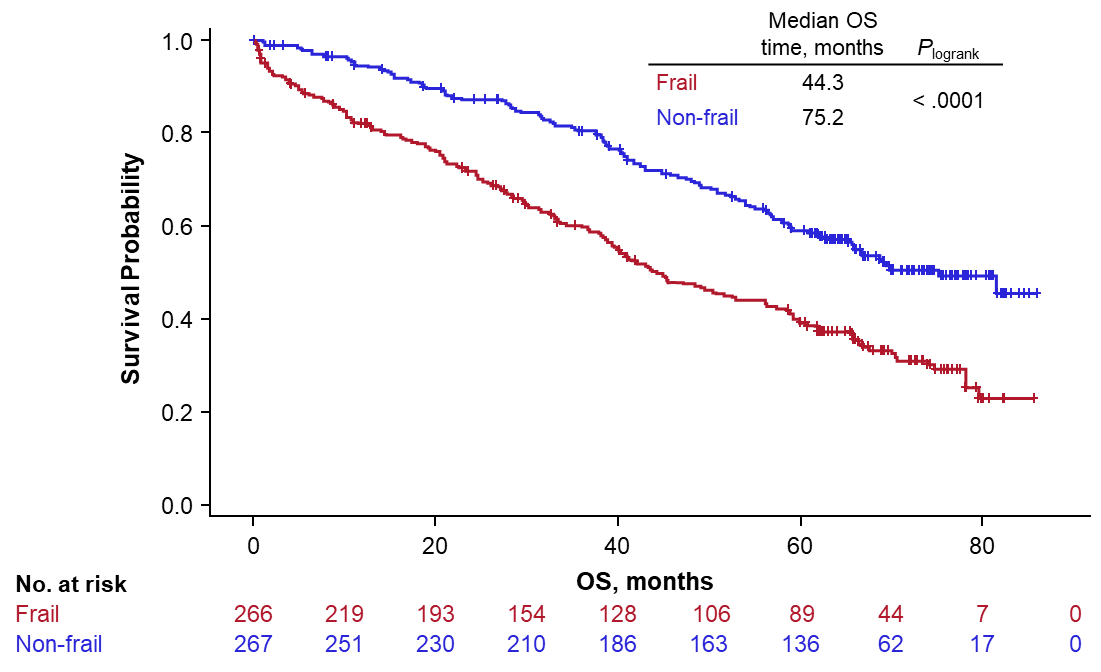
A. B.**

**
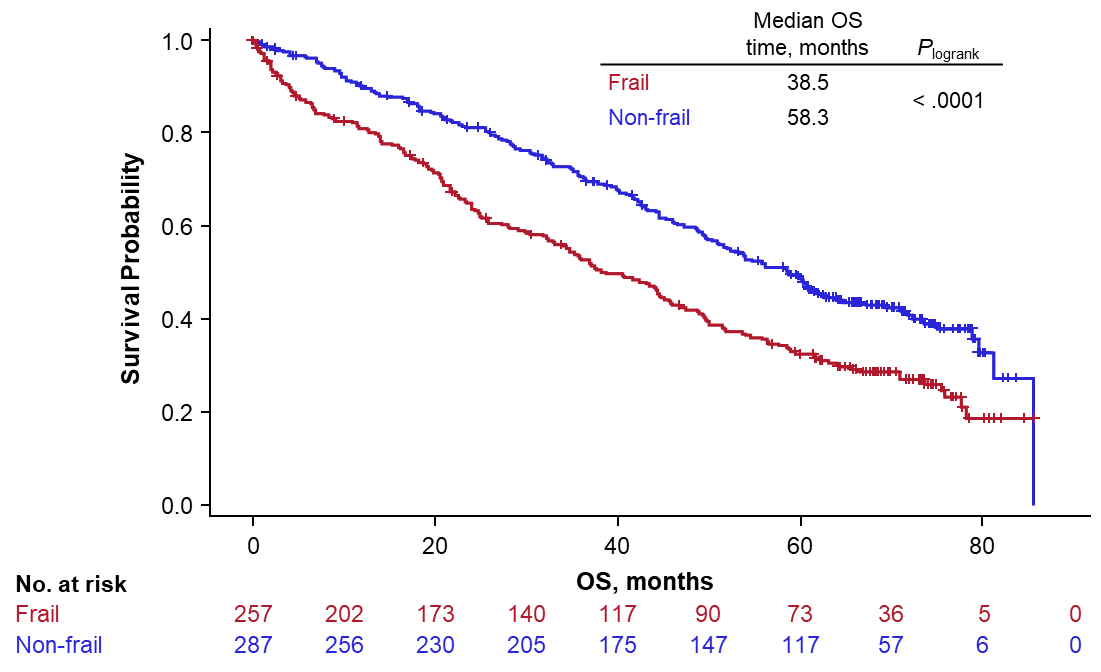
C.**

MPT, melphalan + prednisone + thalidomide; OS, overall survival; Rd continuous, lenalidomide and low-dose dexamethasone until disease progression; Rd18, Rd for 18 cycles.

**Supplemental Figure 3.** Breakdown of frailty and ISS group by treatment arm.

ISS, International Staging System; MPT + melphalan + prednisone + thalidomide; Rd cont, lenalidomide and low-dose dexamethasone until disease progression; Rd18, Rd for 18 cycles.

**
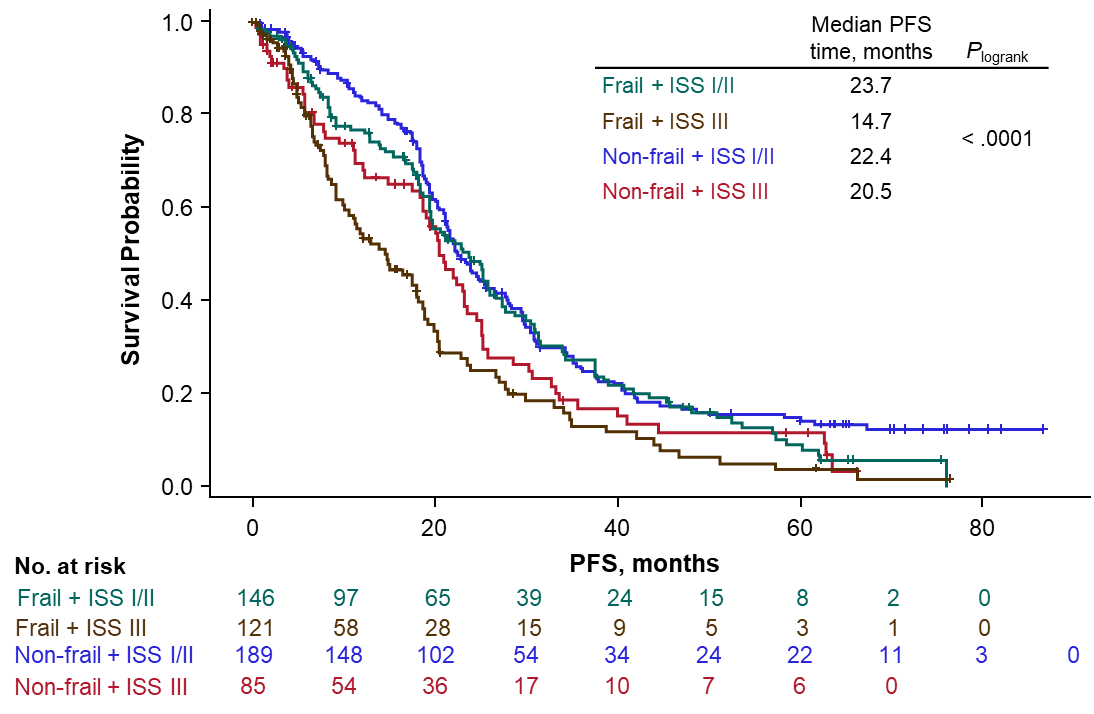
Supplemental Figure 4.** PFS by frailty and ISS group according to treatment arm: Rd continuous **(A)**, Rd18 **(B)**, and MPT **(C)**.^[[11]](#endnote-12)^

**
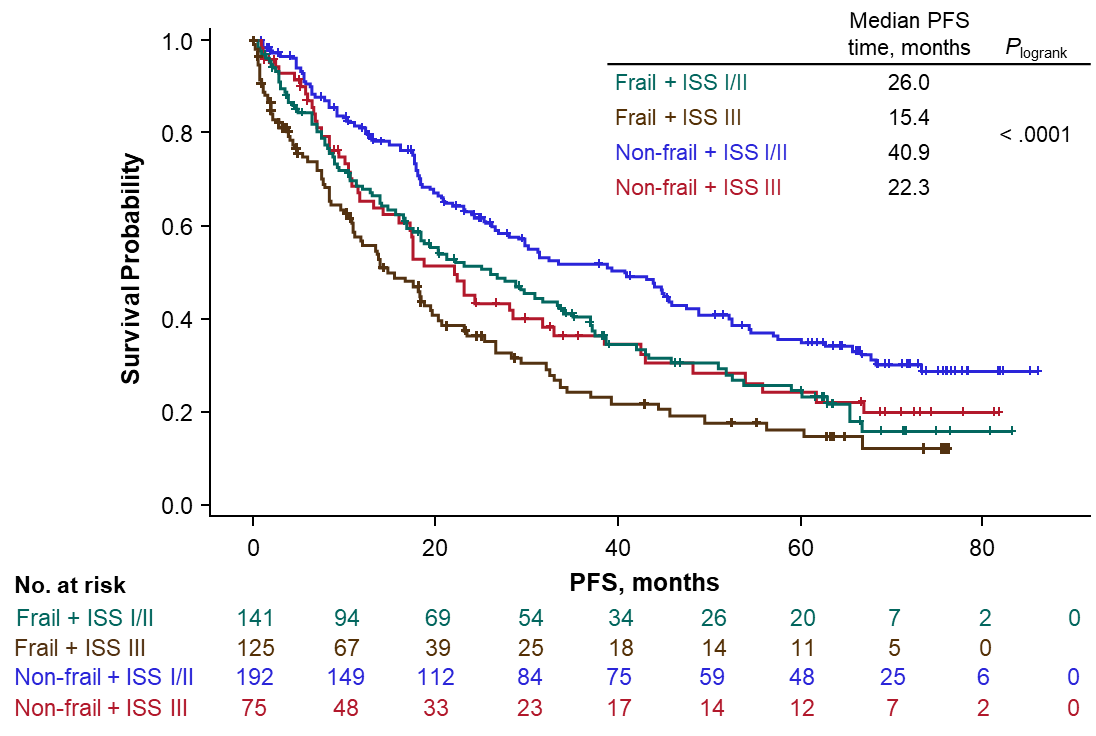
A. B.**

**
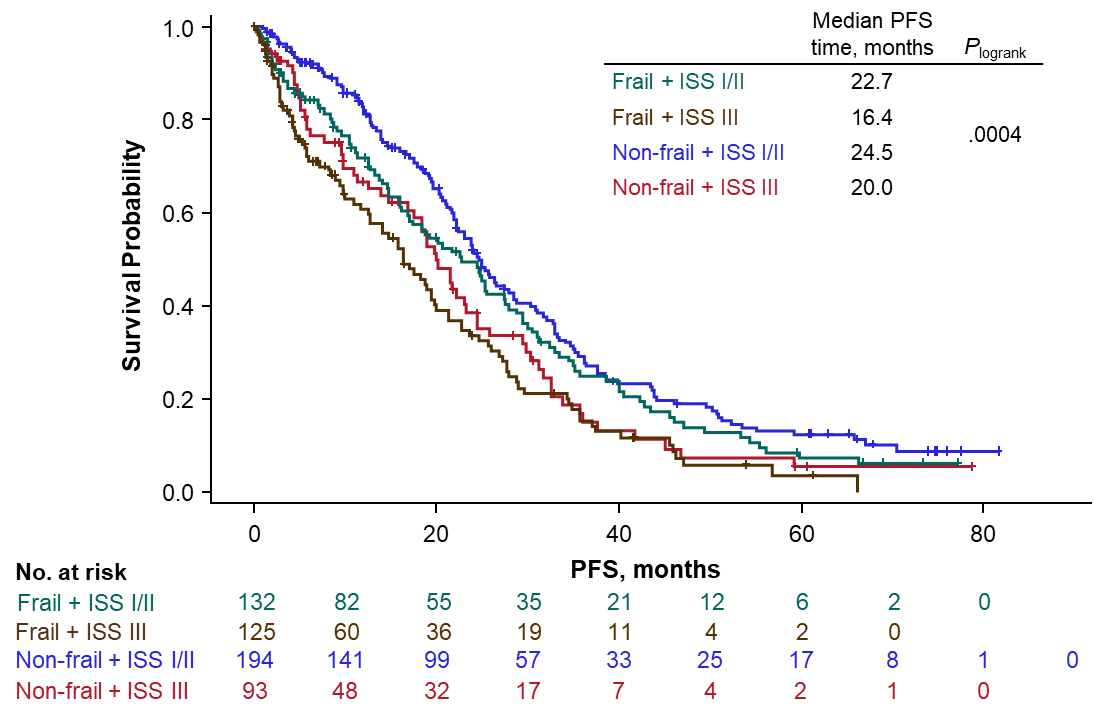
C.**

ISS, International Staging System; MPT, melphalan + prednisone + thalidomide; PFS, progression-free survival; Rd continuous, lenalidomide and low-dose dexamethasone until disease progression; Rd18, Rd for 18 cycles.

**
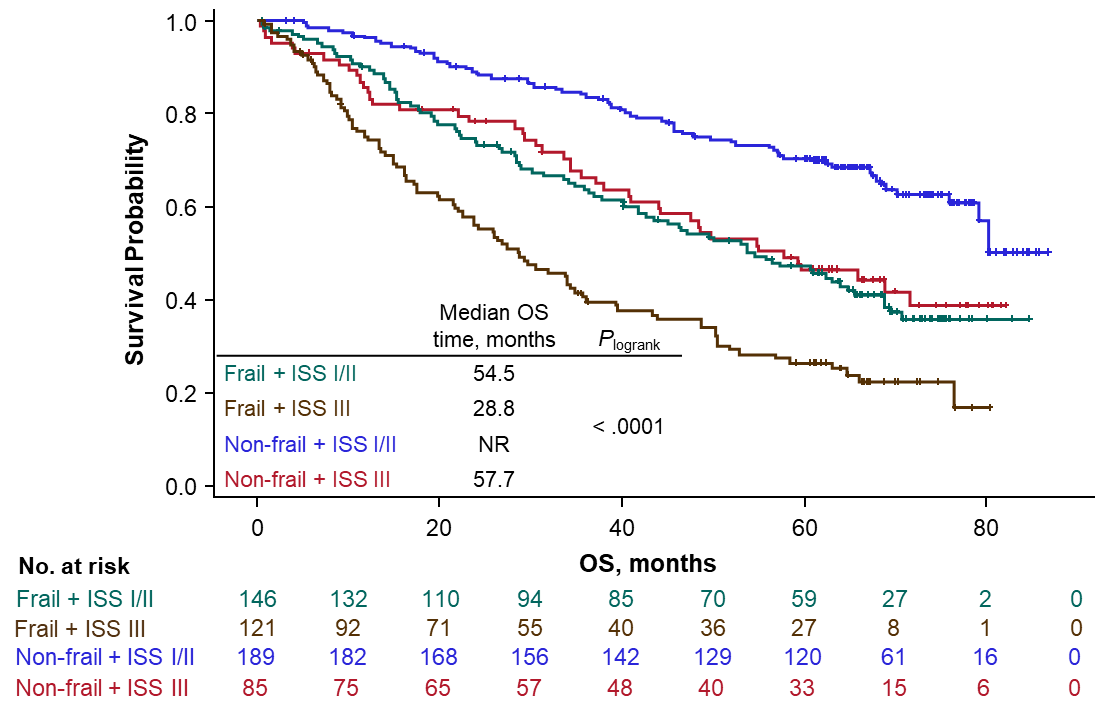
Supplemental Figure 5.** OS by frailty and ISS group according to treatment arm: Rd continuous **(A)**, Rd18 **(B)**, and MPT **(C)**.^[[12]](#endnote-13)^

**A.
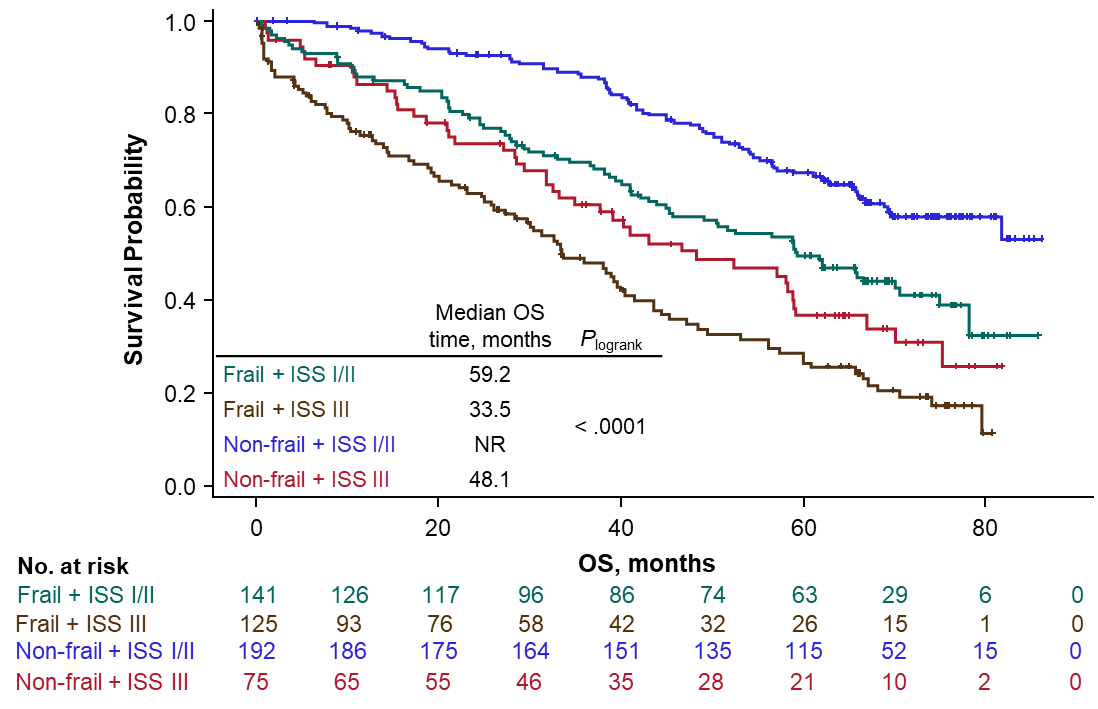
 B.**

**
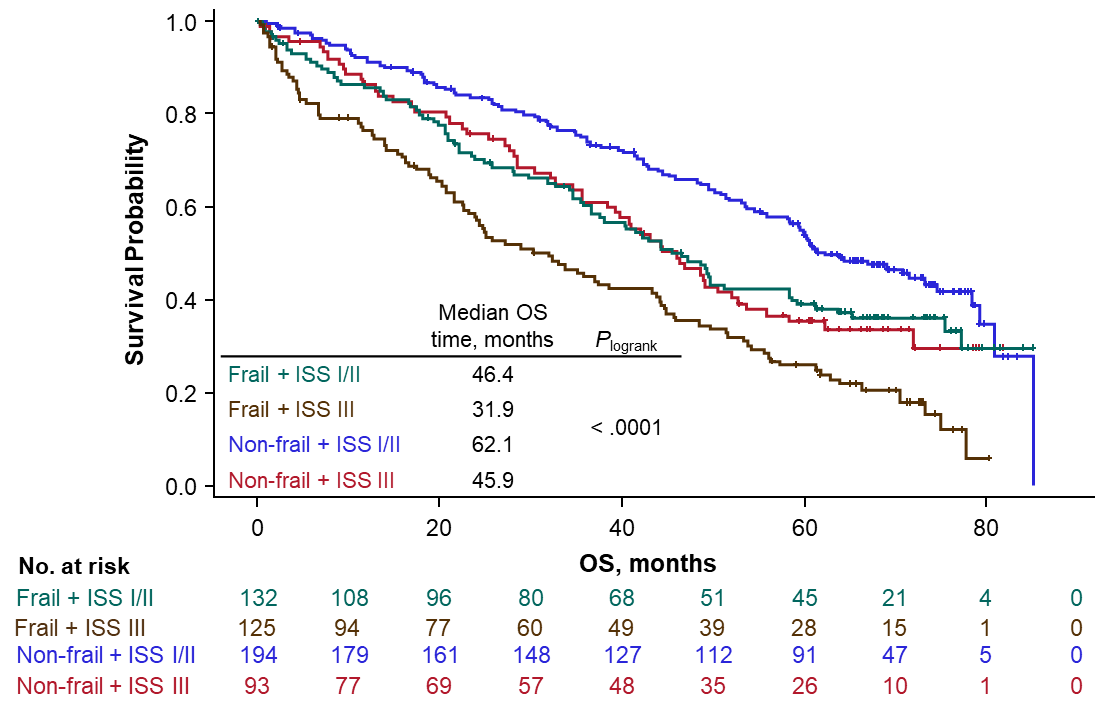
C.**

ISS, International Staging System; MPT, melphalan + prednisone + thalidomide; NR, not reached; OS, overall survival; Rd continuous, lenalidomide and low-dose dexamethasone until disease progression; Rd18, Rd for 18 cycles.

**
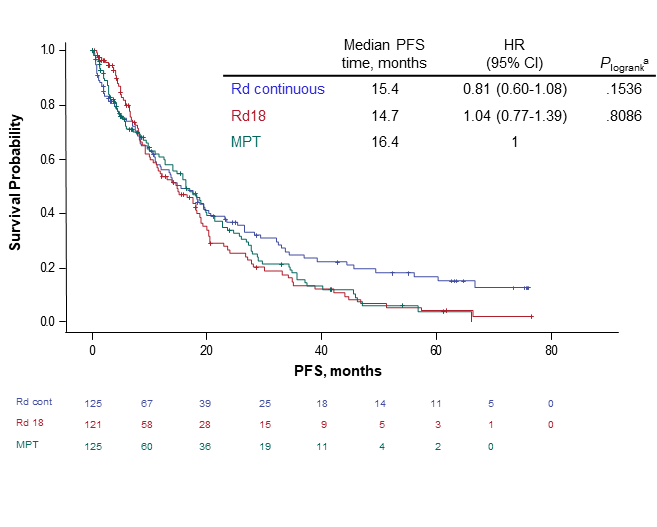
Supplemental Figure 6.** PFS by treatment arm according to frailty and ISS group: frail + ISS I/II **(A)**, frail + ISS III **(B)**, non-frail + ISS I/II **(C)**, and non-frail + ISS III **(D)**.^[[13]](#endnote-14)^

**
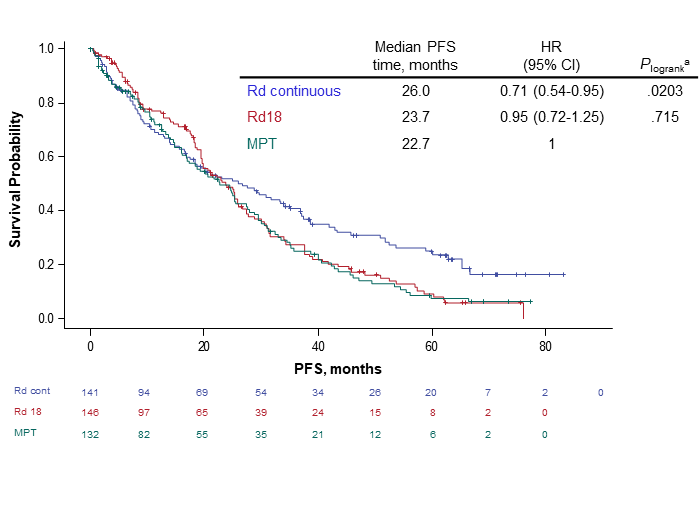
A. B.**

**
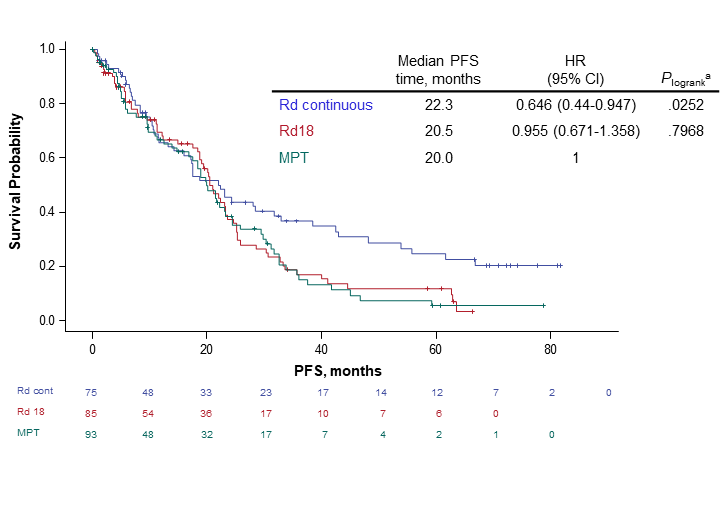

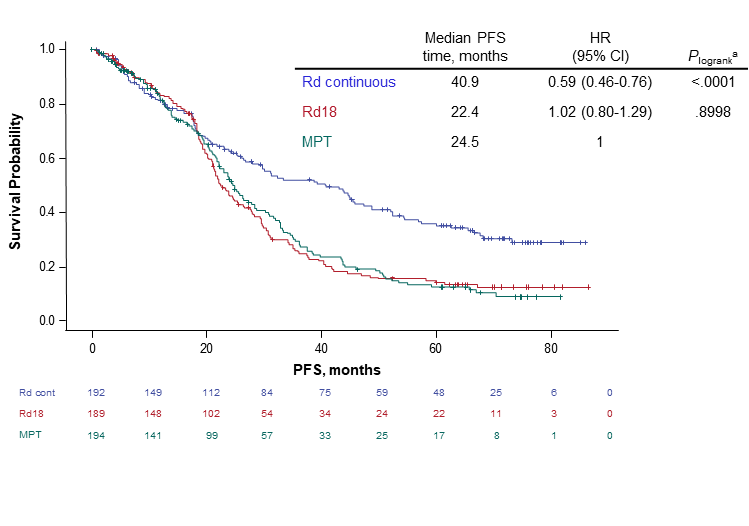
**

**C. D.**

HR, hazard ratio; ISS, International Staging System; MPT, melphalan + prednisone + thalidomide; PFS, progression-free survival; Rd continuous, lenalidomide and low-dose dexamethasone until disease progression; Rd18, Rd for 18 cycles.

^a^ *P* values compare with MPT.

**Supplemental Figure 7.** OS by treatment arm according to frailty and ISS group: frail + ISS I/II **
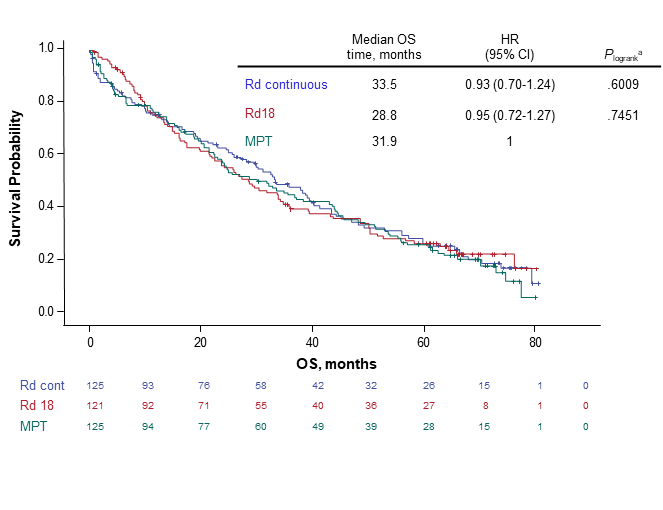

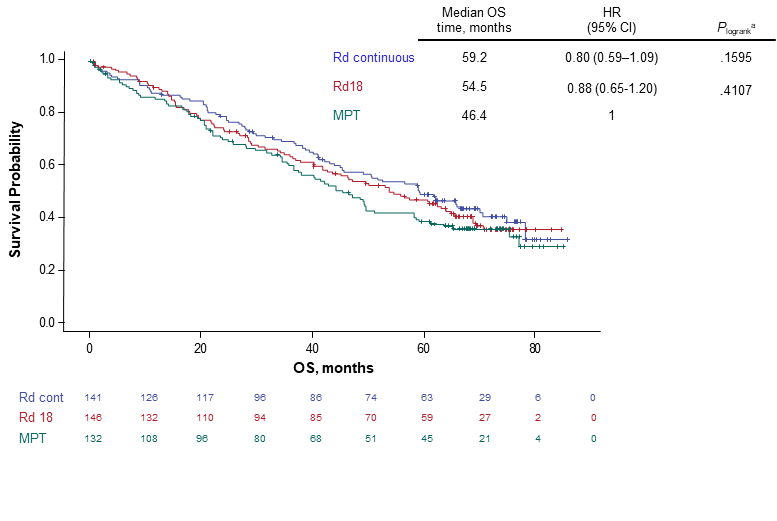
(A)**, frail + ISS III **(B)**, non-frail + ISS I/II **(C)**, and non-frail + ISS III **(D)**.^[[14]](#endnote-15)^

**A. B.**

**
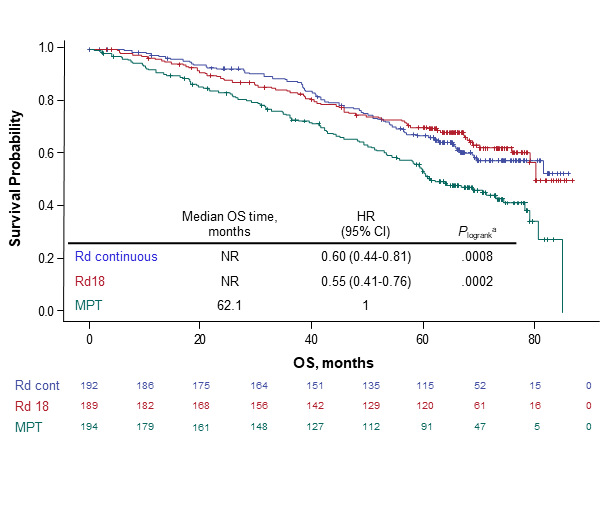
**

**
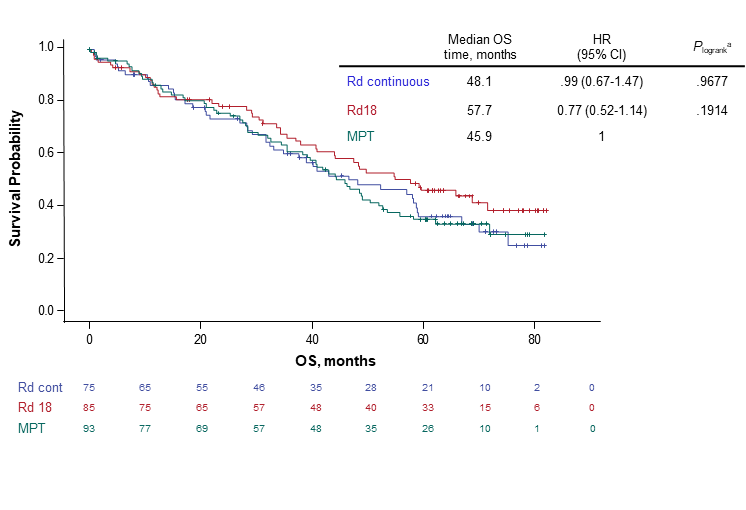
C. D.**

HR, hazard ratio; ISS, International Staging System; MPT, melphalan + prednisone + thalidomide; NR, not reached; OS, overall survival; Rd continuous, lenalidomide and low-dose dexamethasone until disease progression; Rd18, Rd for 18 cycles.

^a^ *P* values compare with MPT.

**Supplemental Table 1.** Response rates by frailty group^[[15]](#endnote-16)^

|  | **Non-Frail**  **(n = 828)** | **Frail**  **(n = 790)** |
| --- | --- | --- |
|  | **n (%)** | |
| Overall response | 658 (79) | 565 (72) |
| CR | 164 (20) | 132 (17) |
| VGPR | 192 (23) | 192 (24) |
| PR | 302 (36) | 241 (31) |
| SD | 121 (15) | 143 (18) |
| PD | 17 (2) | 15 (2) |
| NE | 32 (4) | 67 (8) |

CR, complete response; NE, not evaluable; PD, progressive disease; PR, partial response; SD, stable disease; VGPR, very good partial response.

**Supplemental Table 2.** Summary of grade 3/4 TEAEs by frailty group^[[16]](#endnote-17)^

|  | **Non-Frail (n = 825)** | | | | | | **Frail (n = 783)** | | | | | | **All** | |
| --- | --- | --- | --- | --- | --- | --- | --- | --- | --- | --- | --- | --- | --- | --- |
|  | **Rd18** | | **MPT** | | **Rd** | | **Rd18** | | **MPT** | | **Rd** | |  |  |
|  | **n** | **%** | **n** | **%** | **n** | **%** | **n** | **%** | **n** | **%** | **n** | **%** | **n** | **%** |
| All | 274 | 100 | 285 | 100 | 266 | 100 | 266 | 100 | 253 | 100 | 264 | 100 | 1608 | 100 |
| Neutropenia | 205 | 74.8 | 153 | 53.7 | 186 | 69.9 | 192 | 72.2 | 144 | 56.9 | 189 | 71.6 | 1069 | 66.5 |
| No |  |  |  |  |  |  |  |  |  |  |  |  |  |  |
| Yes | 69 | 25.2 | 132 | 46.3 | 80 | 30.1 | 74 | 27.8 | 109 | 43.1 | 75 | 28.4 | 539 | 33.5 |
| Anemia | 241 | 88.0 | 230 | 80.7 | 222 | 83.5 | 214 | 80.5 | 207 | 81.8 | 209 | 79.2 | 1323 | 82.3 |
| No |  |  |  |  |  |  |  |  |  |  |  |  |  |  |
| Yes | 33 | 12.0 | 55 | 19.3 | 44 | 16.5 | 52 | 19.5 | 46 | 18.2 | 55 | 20.8 | 285 | 17.7 |
| Thrombocytopenia | 253 | 92.3 | 246 | 86.3 | 248 | 93.2 | 244 | 91.7 | 233 | 92.1 | 234 | 88.6 | 1458 | 90.7 |
| No |  |  |  |  |  |  |  |  |  |  |  |  |  |  |
| Yes | 21 | 7.7 | 39 | 13.7 | 18 | 6.8 | 22 | 8.3 | 20 | 7.9 | 30 | 11.4 | 150 | 9.3 |
| Leukopenia | 260 | 94.9 | 258 | 90.5 | 250 | 94.0 | 250 | 94.0 | 227 | 89.7 | 255 | 96.6 | 1500 | 93.3 |
| No |  |  |  |  |  |  |  |  |  |  |  |  |  |  |
| Yes | 14 | 5.1 | 27 | 9.5 | 16 | 6.0 | 16 | 6.0 | 26 | 10.3 | 9 | 3.4 | 108 | 6.7 |
| Infection | 221 | 80.7 | 242 | 84.9 | 185 | 69.5 | 201 | 75.6 | 204 | 80.6 | 177 | 67.0 | 1230 | 76.5 |
| No |  |  |  |  |  |  |  |  |  |  |  |  |  |  |
| Yes | 53 | 19.3 | 43 | 15.1 | 81 | 30.5 | 65 | 24.4 | 49 | 19.4 | 87 | 33.0 | 378 | 23.5 |
| Cardiac disorders | 257 | 93.8 | 266 | 93.3 | 236 | 88.7 | 244 | 91.7 | 226 | 89.3 | 227 | 86.0 | 1456 | 90.5 |
| No |  |  |  |  |  |  |  |  |  |  |  |  |  |  |
| Yes | 17 | 6.2 | 19 | 6.7 | 30 | 11.3 | 22 | 8.3 | 27 | 10.7 | 37 | 14.0 | 152 | 9.5 |
| Fatigue | 252 | 92.0 | 271 | 95.1 | 244 | 91.7 | 242 | 91.0 | 236 | 93.3 | 244 | 92.4 | 1489 | 92.6 |
| No |  |  |  |  |  |  |  |  |  |  |  |  |  |  |
| Yes | 22 | 8.0 | 14 | 4.9 | 22 | 8.3 | 24 | 9.0 | 17 | 6.7 | 20 | 7.6 | 119 | 7.4 |
| Back pain | 255 | 93.1 | 272 | 95.4 | 246 | 92.5 | 251 | 94.4 | 238 | 94.1 | 245 | 92.8 | 1507 | 93.7 |
| No |  |  |  |  |  |  |  |  |  |  |  |  |  |  |
| Yes | 19 | 6.9 | 13 | 4.6 | 20 | 7.5 | 15 | 5.6 | 15 | 5.9 | 19 | 7.2 | 101 | 6.3 |
| Peripheral sensory neuropathy | 273 | 99.6 | 252 | 88.4 | 261 | 98.1 | 265 | 99.6 | 235 | 92.9 | 263 | 99.6 | 1549 | 96.3 |
| No |  |  |  |  |  |  |  |  |  |  |  |  |  |  |
| Yes | 1 | 0.4 | 33 | 11.6 | 5 | 1.9 | 1 | 0.4 | 18 | 7.1 | 1 | 0.4 | 59 | 3.7 |
| Cataract | 262 | 95.6 | 282 | 98.9 | 245 | 92.1 | 264 | 99.2 | 253 | 100 | 248 | 93.9 | 1554 | 96.6 |
| No |  |  |  |  |  |  |  |  |  |  |  |  |  |  |
| Yes | 12 | 4.4 | 3 | 1.1 | 21 | 7.9 | 2 | 0.8 | 0 | 0 | 16 | 6.1 | 54 | 3.4 |
| DVT | 264 | 96.4 | 277 | 97.2 | 249 | 93.6 | 256 | 96.2 | 247 | 97.6 | 252 | 95.5 | 1545 | 96.1 |
| No |  |  |  |  |  |  |  |  |  |  |  |  |  |  |
| Yes | 10 | 3.6 | 8 | 2.8 | 17 | 6.4 | 10 | 3.8 | 6 | 2.4 | 12 | 4.5 | 63 | 3.9 |
| PE | 265 | 96.7 | 271 | 95.1 | 255 | 95.9 | 259 | 97.4 | 247 | 97.6 | 255 | 96.6 | 1552 | 96.5 |
| No |  |  |  |  |  |  |  |  |  |  |  |  |  |  |
| Yes | 9 | 3.3 | 14 | 4.9 | 11 | 4.1 | 7 | 2.6 | 6 | 2.4 | 9 | 3.4 | 56 | 3.5 |
| Invasive SPM | 254 | 92.7 | 260 | 91.2 | 243 | 91.4 | 248 | 93.2 | 232 | 91.7 | 251 | 95.1 | 1488 | 92.5 |
| No |  |  |  |  |  |  |  |  |  |  |  |  |  |  |
| Yes | 20 | 7.3 | 25 | 8.8 | 23 | 8.6 | 18 | 6.8 | 21 | 8.3 | 13 | 4.9 | 120 | 7.5 |
| Hematologic malignancies | 272 | 99.3 | 276 | 96.8 | 262 | 98.5 | 266 | 100 | 248 | 98.0 | 264 | 100 | 1588 | 98.8 |
| No |  |  |  |  |  |  |  |  |  |  |  |  |  |  |
| Yes | 2 | 0.7 | 9 | 3.2 | 4 | 1.5 | 0 | 0 | 5 | 2.0 | 0 | 0 | 20 | 1.2 |
| Solid tumor | 255 | 93.1 | 269 | 94.4 | 247 | 92.9 | 248 | 93.2 | 237 | 93.7 | 251 | 95.1 | 1507 | 93.7 |
| No |  |  |  |  |  |  |  |  |  |  |  |  |  |  |
| Yes | 19 | 6.9 | 16 | 5.6 | 19 | 7.1 | 18 | 6.8 | 16 | 6.3 | 13 | 4.9 | 101 | 6.3 |
| Noninvasive SPM (NMSC) | 260 | 94.9 | 268 | 94.0 | 250 | 94.0 | 253 | 95.1 | 240 | 94.9 | 248 | 93.9 | 1519 | 94.5 |
| No |  |  |  |  |  |  |  |  |  |  |  |  |  |  |
| Yes | 14 | 5.1 | 17 | 6.0 | 16 | 6.0 | 13 | 4.9 | 13 | 5.1 | 16 | 6.1 | 89 | 5.5 |

DVT, deep vein thrombosis; MPT, melphalan + prednisone + thalidomide; NMSC, non-melanoma skin cancer; PE, pulmonary embolism; Rd, lenalidomide and low-dose dexamethasone; Rd18, Rd for 18 cycles; SPM, secondary primary malignancy; TEAE, treatment-emergent adverse event.

**Supplemental Table 3.** Baseline characteristics by frailty and ISS stage^[[17]](#endnote-18)^

| **Characteristic** | **Non-Frail +  ISS I/II^a^**  **(n = 575)** | **Non-Frail +  ISS III^a^**  **(n = 253)** | **Frail +  ISS I/II^a^**  **(n = 419)** | **Frail +  ISS III^a^**  **(n = 371)** |
| --- | --- | --- | --- | --- |
| Age, n (%) | | | | |
| Median (range), years | 70 (40-80) | 71 (43-80) | 76 (44-91) | 77 (53-92) |
| < 65 years | 43 (7) | 23 (9) | 16 (4) | 10 (3) |
| 65-75 years | 481 (83) | 207 (82) | 151 (36) | 122 (33) |
| 76-80 years | 51 (9) | 23 (9) | 154 (37) | 136 (37) |
| > 80 years | 0 | 0 | 98 (23) | 103 (28) |
| Sex, n (%) | | | | |
| Male  Female | 305 (53)  270 (47) | 131 (52)  122 (48) | 206 (49)  213 (51) | 209 (56)  162 (44) |
| ECOG performance status, n (%)^b^ | | | | |
| 0 | 290 (50) | 111 (44) | 43 (10) | 30 (8) |
| 1 | 285 (50) | 142 (56) | 190 (45) | 178 (48) |
| 2 | 0 | 0 | 184 (44) | 159 (43) |
| 3 | 0 | 0 | 2 (< 1) | 4 (1) |
| Lactate dehydrogenase, n (%) | | | | |
| < 200 U/L | 510 (89) | 197 (78) | 348 (83) | 264 (71) |
| ≥ 200 U/L | 64 (11) | 56 (22) | 71 (17) | 106 (29) |
| Missing data | 1 (< 1) | 0 | 0 | 1 (< 1) |
| Creatinine clearance, n (%) | | | | |
| < 60 mL/min | 146 (25) | 153 (60) | 173 (41) | 305 (82) |
| < 30 mL/min | 8 (1) | 30 (12) | 14 (3) | 94 (25) |
| ≥ 60 mL/min | 429 (75) | 100 (40) | 246 (59) | 66 (18) |
| ECOG, Eastern Cooperative Oncology Group; ISS, International Staging System.  ^a^ Higher stages indicate more severe disease.  ^b^ ECOG scores range from 0 to 5, with higher numbers indicating greater disability. | | | | |

**Supplemental Table 4.** Dose intensity by frailty group^a^^[[18]](#endnote-19)^

| **Dose Intensity** | **Non-Frail (n = 828)** | **Frail (n = 790)** |
| --- | --- | --- |
| Rd | | |
| Lenalidomide  n  Median (range), mg/day | 540  16.2 (1.5-19.6) | 530  11.9 (1.9-25.0) |
| Dexamethasone  n  Median (range), mg/ncy | 540  4.7 (0.2-6.2) | 530  2.8 (0.8-7.5) |
| MPT | | |
| Melphalan  n  Median (range), mg/day | 284  1.2 (0.2-5.6) | 253  0.9 (0.1-10.7) |
| Prednisone  n  Median (range), mg/day | 285  12.8 (4.2-65.7) | 253  12.4 (3.9-108.8) |
| Thalidomide  n  Median (range), mg/day | 284  134.1 (24.4-200.0) | 253  92.3 (6.7-200.0) |

^a^ Dexamethasone, melphalan, prednisone, and thalidomide had starting doses that were specified by age; therefore, the medians may be impacted.

1. FXCX: MM020 frailty assessment (nonfrail vs frail) 21Nov2016 Batch 1, slide 12 [↑](#endnote-ref-2)
2. FXCX: MM020 frailty assessment (nonfrail vs frail) 21Nov2016 Batch 1, slide 12 [↑](#endnote-ref-3)
3. FXCX: MM020 frailty assessment (nonfrail vs frail) 21Nov2016 Batch 1, slide 10 [↑](#endnote-ref-4)
4. FXCX: MM020 frailty assessment (nonfrail vs frail) 21Nov2016 Batch 1, slide 16 [↑](#endnote-ref-5)
5. FXCX: MM020 frailty assessment (nonfrail vs frail) 21Nov2016 Batch 1, slide 16 [↑](#endnote-ref-6)
6. FXCX: MM020 frailty assessment (nonfrail vs frail) 21Nov2016 Batch 1, slide 14 [↑](#endnote-ref-7)
7. FXCX: MM020 frailty assessment (nonfrail vs frail) 21Nov2016 Batch 1, slide 19-20 [↑](#endnote-ref-8)
8. FXCX: MM020 frailty assessment (nonfrail vs frail) 21Nov2016 Batch 1, slide 23-24 [↑](#endnote-ref-9)
9. FXCX: MM020 frailty assessment (nonfrail vs frail) 21Nov2016 Batch 1, slides 10 [↑](#endnote-ref-10)
10. FXCX: MM020 frailty assessment (nonfrail vs frail) 21Nov2016 Batch 1, slides 14 [↑](#endnote-ref-11)
11. FXCX: MM020 frailty assessment (nonfrail vs frail) 21Nov2016 Batch 1, slide 41 [↑](#endnote-ref-12)
12. FXCX: MM020 frailty assessment (nonfrail vs frail) 21Nov2016 Batch 1, slide 44 [↑](#endnote-ref-13)
13. FXCX: MM020 frailty assessment (nonfrail vs frail) 21Nov2016 Batch 1, slide 41 [↑](#endnote-ref-14)
14. FXCX: MM020 frailty assessment (nonfrail vs frail) 21Nov2016 Batch 1, slide 44 [↑](#endnote-ref-15)
15. FXCX: For Overall response, MM020 frailty assessment (nonfrail vs frail) 21Nov2016 Batch 1, slide 31 & 30; For rest of table, MM020 frailty assessment (nonfrail vs frail) 21Nov2016 Batch 1, slide 30 [↑](#endnote-ref-16)
16. FXCX: Summary Gr 3_4 TEAEs or SPM By Dichotomized Frailty ECOG IMWG - Safety Pop v3_0 [↑](#endnote-ref-17)
17. FXCX: MM020 frailty assessment (nonfrail vs frail) 21Nov2016 Batch 1, slide 39 [↑](#endnote-ref-18)
18. FXCX: MM020 frailty assessment (nonfrail vs frail) 14Nov2016 Batch 2, slide 17 [↑](#endnote-ref-19)
